# The clinical significance and potential therapeutic target of tumor-associated macrophage in non-small cell lung cancer

**DOI:** 10.3389/fmed.2025.1541104

**Published:** 2025-04-30

**Authors:** Jiazheng Sun, Sirui Zhou, Yalu Sun, Yulan Zeng

**Affiliations:** ^1^Liyuan Hospital, Tongji Medical College, Huazhong University of Science and Technology, Wuhan, China; ^2^Affiliated Hospital of Jining Medical University, Jining, China

**Keywords:** tumor-associated macrophage, non-small cell lung cancer, therapeutic targets, immune escape, tumor microenvironment

## Abstract

One of the leading causes of cancer-related mortality globally is non-small cell lung cancer (NSCLC). It has become a significant public health concern due to its rising incidence rate and fatality. Tumor-associated macrophage (TAM) is important in the tumor microenvironment (TME) of NSCLC because they have an impact on the development, metastasis, and incidence of tumors. As a crucial element of the TME, TAM contributes to tumor immune evasion, facilitates tumor proliferation and metastasis, and modulates tumor angiogenesis, immunosuppression, and treatment resistance through the secretion of diverse cytokines, chemokines, and growth factors. Consequently, TAM assumes a multifaceted and intricate function in the onset, progression, and therapeutic response of NSCLC, serving as a crucial focal point for comprehending the tumor microenvironment and formulating novel therapeutic methods. The study aims to review the biological properties and potential processes of TAM in NSCLC, investigate its involvement in the clinical of NSCLC patients, and discuss its potential as a therapeutic target.

## 1 Introduction

Lung cancer is one of the leading causes of cancer death worldwide (1). Non-small cell lung cancer (NSCLC) is the most common type of lung cancer, and according to the study, NSCLC patients account for about 80–85% of all lung cancer patients (2). In addition, with the increase in risk factors such as smoking, air pollution, and chronic obstructive pulmonary disease, the incidence of NSCLC is increasing globally (3). These factors not only affect the survival rate of patients but also pose serious challenges to public health.

Tumor cells, stromal cells, blood vessels, and immune cells are some of the components that make up the tumor microenvironment (TME), which plays a crucial role in tumor growth and metastasis (4). Through signal transduction and intercellular interactions, TME influences tumor growth and responsiveness to therapy (5). The macrophage present in TME is called tumor-associated macrophage (TAM). While TAM can promote tumor development and spread by creating an immune escape route for malignancies, it can also prevent tumor growth by engulfing tumor cells and secreting cytokines (6).

The purpose of the study is to review the role and subclassification of TAM in NSCLC and discuss its potential as a therapeutic target. The upcoming chapters will cover these subjects in detail, to offer references for future studies and clinical applications.

## 2 The origin of TAM

In most solid tumors, TAM and their precursors constitute the largest proportion of bone marrow infiltration among the cell types associated with the TME (7). 50% of the tumor mass may be made up of them (8, 9). The origin of TAM represents complex; They primarily derive from peripheral blood monocytes that circulate to the TME and differentiate into TAM in the presence of local cytokines (10). Besides, certain tumors may utilize pre-existing tissue-resident macrophages located in adipose tissue, liver, or lungs, which may undergo phenotypic changes within the TME (11). Additionally, tumor cells can secrete various cytokines and chemokines, such as CCL2 and CSF-1, which promote the migration of monocytes to the tumor site and facilitate their polarization into different types of TAM (12).

## 3 The TAM subclassification

### 3.1 Classical TAM subclassification

**Figure 1:**
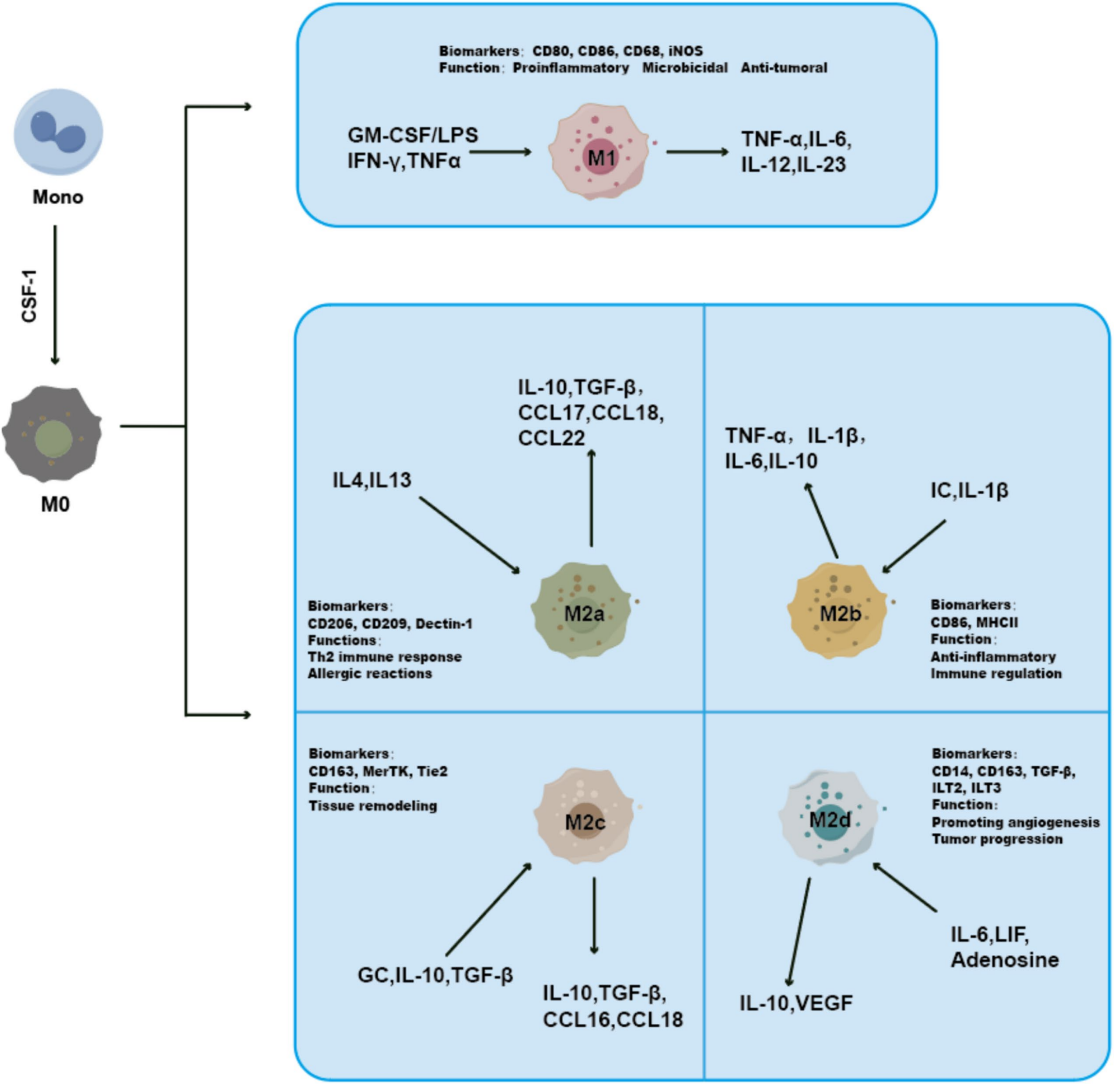
CSF-1 could induce monocytes to differentiate into M0 macrophages. Then, M0 macrophages could further evolve into M1 or M2 macrophages stimulated by Th1-type or Th2-type cytokines. Due to their differences in activation patterns and other aspects, M2 macrophages could be further divided into four subtypes: M2a, M2b, M2c, and M2d. colony-stimulating factor 1, CSF-1; interferon-*γ*, IFN-γ; lipopolysaccharides, LPS; granulocyte monocyte colony-stimulating factor, GM-CSF; tumor necrosis factor-*α*, TNF-α; immune complexes, IC; C-C motif chemokine 17, CCL17; interleukin-4 IL-4; glucocorticoid, GC; leukemia inhibitory factor, LIF; transforming growth factor-β, TGF-β.

The concept of M1-TAM and M2-TAM (Figure 1) was initially introduced by Mills et al. (13). The biomarkers CD80, CD86, CD68, and iNOS are up-regulatedly expressed in M1-TAM, which mostly secretes pro-inflammatory cytokines such as TNF-*α*, IL-6, IL-12, and IL-23 (14). M1-TAM polarization is typically triggered by T helper type 1-associated cytokines, including IFN-*γ*, TNF-α, LPS, and GM-CSF (15, 16). M1-TAM can limit tumor cell proliferation and augment the anti-tumor immune response by facilitating T cell activation and proliferation in the TME (17).

M2-TAM plays a crucial immunomodulatory role in the TME. The M2-TAM subclassifications are classified into M2a-TAM, M2b-TAM, M2c-TAM, and M2d-TAM based on their specific roles and phenotypic characteristics. The biomarkers CD206, CD209, and Dectin-1 are up-regulatedly expressed in M2a-TAM, which is primarily involved in Th2 immune response. M2a-TAM polarization is typically triggered by IL-4 and IL-13 (18). The biomarkers CD86 and MHC II are up-regulatedly expressed in M2b-TAM, which primarily contributes to the anti-inflammatory and immune regulation by secreting cytokines like TNF-*α*, IL-6, and IL-10. M2b-TAM polarization is typically triggered by IC and IL-1*β*. M2c-TAM polarization is typically triggered by GC, TGF-β, and IL-10 (19). The biomarkers CD163, MerTK, and Tie2 are up-regulatedly expressed in M2c-TAM, while CD14, CD86, CD16, and CD206 are down-regulatedly expressed at low levels (20–22). M2c-TAM primarily contributes to tissue remodeling (19). M2d-TAM polarization is typically triggered by adenosine, LIF, and IL-6. M2d-TAM mostly contributes to stimulating angiogenesis and extracellular matrix disintegration to promote tumor spread, with up-regulated expression levels of biomarkers CD14, CD163, ILT2, and ILT3 (23).

In conclusion, the different polarization states of M1-TAM and M2-TAM play various roles in the TME. M1-TAM generally appears as a promoter of anti-tumor immunity, while M2-TAM, through its different subtypes, plays a role in immunosuppression, tissue remodeling, and promoting tumor development.

### 3.2 TAM subclassification based on scRNAseq information

The increasing application of scRNAseq information in cancer research is enhancing the understanding of tumor biology. scRNAseq information aids in identifying distinct cell subsets and their characteristics, while also revealing cell heterogeneity within tumors. This is particularly important for analyzing the TME and understanding tumor development and metastasis. Seven TAM subtypes are present in nearly all malignancies (Table 1), according to recent widely used scRNAseq analysis studies. These TAM subgroups are named interferon-induced TAM (IFN-TAM), immunomodulatory TAM (Reg-TAM), inflammatory cytokine enriched TAM (Inflam-TAM), lipid-associated TAM (LA-TAM), angiogenic TAM (AngioTAM), tissue-resident TAM (RTM-TAM), and proliferative TAM (Prolif-TAM) based on their signature genes, enrichment pathways, and potential roles (24).

**Table 1:** TAM subclassification based on scRNAseq information.

TAM Type	Markers	Properties/Functions
IFN-TAM	CXCL10, PDL1, ISG15	Immunosuppressive characteristics; High expression of interferon regulatory genes, similar to M1-TAM
Reg-TAM	CX3CR1, MRC1, TAMARG1	Possible immunosuppressive properties; further research needed to determine its specific functions
Inflam-TAM	IL1B, CXCL1/2/3/8, CCL3, CCL3L1	Involved in immune cell recruitment and regulation in the inflammatory response associated with tumors
Angio-TAM	VEGFA, SPP1	Promotes angiogenesis and metastasis, contributing to tumor development
LA-TAM	APOC1, APOE, ACP5, FABP5	Active suppression of anti-tumor immune response; lipid-related gene expression
RTM-TAM	LYVE1, HES1, FOLR	May promote tumor invasion and progression, similar to normal tissue TAM
Prolif-TAM	MKI67, Cell cycle-related genes	Potential pro-inflammatory functions; contribute to tumor progression

IFN-TAM demonstrates immunosuppressive characteristics while also exhibiting high expression of interferon regulatory genes, including CXCL10, PDL1, and ISG15, similar to M1-TAM (25, 26). Reg-TAM exhibits high expression levels of CX3CR1, MRC1, and TAMARG1. It may possess immunosuppressive properties; however, further research is required to determine its specific functions (27–29). Inflammatory cytokines such as IL1B, CXCL1/2/3/8, CCL3, and CCL3L1 are expressed by Inflam-TAM (25, 30), which is involved in immune cell recruitment and regulation in the inflammatory response associated with tumors (31). By encouraging angiogenesis and metastasis, angiogenic markers, such as VEGFA and SPP1, contribute to the development of tumors, which are highly expressed in the Angio-TAM (25, 30, 32, 33). Lipid-related genes, including APOC1, APOE, ACP5, and FABP5, are highly expressed by LA-TAM and demonstrated active suppression of the anti-tumor immune response (30, 34, 35). RTM-TAM is similar to normal tissue TAM and expresses LYVE1, HES1, and FOLR, which may play a promoting role in tumor invasion and progression (36, 37). The characteristic genes of Prolif-TAM include MKI67 and cell cycle-related genes, which have potential pro-inflammatory functions and play a role in tumor progression (38).

The increasing use of scRNAseq in cancer research is improving the understanding of tumor biology by identifying distinct TAM subclassifications, each with unique gene signatures and roles in tumor development, immune response, and metastasis.

## 4 Signaling crosstalk between TAM and non-tumor cells in TME

### 4.1 TAM and CAF

Fibroblast is an essential biological constituent of the TME, with distinct activities and functions between normal and tumor tissues. Normal fibroblast predominantly contributes to the maintainment of tissue architecture and functionality, however, in tumors, it differentiates into cancer-associated fibroblast (CAF), which displays an activated phenotype and facilitates tumor proliferation and dissemination (39). CAF is differentiated from fibroblasts by its contractile properties, metabolic processes, and transcriptomic activity (40, 41). Furthermore, they exhibited elevated levels of FAP, αSMA, and vimentin (42, 43).

In the TME, the interaction between CAF and TAM plays an important role in tumor progression. On the one hand, CAF secrete CXCL12 to regulate M2-TAM polarization (44, 45). In addition, CAFs can also secrete IL-11 to activate the AXL-STAT3 signal transduction pathway, thereby up-regulating M2-TAM polarization (46). TAM, on the other hand, promotes the activation of CAF and enhances the aggressiveness of tumor cells by producing molecules such as IL-6, CXCL12, and TGF-*β*. In addition, it has been shown that CAF can be derived from M2-TAM, which is called macrophage mesenchymal transformation (MMT). Meanwhile, Smad3 has been found to play a key role in MMT in NSCLC (47).

These complex intercellular signal transduction crosstalk significantly influences the immune environment and metastatic ability of tumors. Therefore, understanding the interaction of CAF and TAM in TME and their signaling pathways is of great significance for tumor therapy.

### 4.2 TAM and MSC

Mesenchymal stromal cell (MSC) is an undifferentiated, adherent stromal cell present in several organs, frequently located at injury sites and within malignancies (48). MSC can facilitate tumorigenic processes, encompassing tumor tissue creation, maintenance, chemotherapy resistance, and tumor proliferation (48, 49). Recent research indicates that substantial functional interactions may occur between MSC and TAM. Babazadeh et al. discovered that the MSC-derived CXCL12 niche affects TAM polarization dynamics by promoting the phenotypic transformation of BMDMs into M2-TAM, potentially playing a crucial role in the TME (44). Furthermore, Ren et al. demonstrated that extracellular vesicles released by hypoxia-preconditioned MSC enhance NSCLC cell proliferation and motility, as well as M2-TAM polarization, through the transfer of miR-21-5p (50). The investigation of the interplay between MSC and TAM will advance the development of innovative cell treatments for cancer.

### 4.3 TAM and other cell

Moreover, certain immune cells regulate TAM polarization within the TME by cytokine secretion, consequently influencing immune evasion, metastasis, and treatment resistance of tumors. The miR-320a, released by neutrophils, down-regulates STAT4 upon entering macrophages and facilitates M2-TAM polarization (51). In addition, the expression of signal regulatory protein-*α* (52) in neutrophils enhances the SHP-1/p38/MAPK/STAT3 signaling pathway and induces M2-TAM polarization (52). IL-17A/IL-17AF released by Th17 cells facilitates NSCLC metastases through the induction of M2-TAM polarization (53). TNFSF15, produced by vascular endothelial cells, inhibits STAT6, resulting in the induction of M1-TAM polarization (54). These pathways offer novel targets and therapeutic approaches for cancer treatment.

## 5 The function of TAM in NSCLC

### 5.1 Reprogramming metabolism

Interactions between tumor cells and surrounding cells cause metabolic reprogramming of TME, which impacts tumor development, metastasis, and immune escape. TME is characterized by hypoxia and lactic acid accumulation (55). The absence of oxygen in the TME and the acidic environment alter the metabolism of immune cells and tumor cells, which encourages immune escape and tumor growth (56).

In the TAM of NSCLC, hypoxia can enhance the tumor-supportive role of TAM by boosting iron availability through the upregulation of associated proteins and facilitating the proliferation of malignant cells (7). Furthermore, TAM will release a range of metabolic cytokines, including IL6, TNF-*α*, and others, in response to hypoxia or a lactic acid environment. These cytokines can encourage the glycolysis of tumor cells (56). TAM increases the intake and synthesis of fatty acids while simultaneously supplying energy for the TCA cycle through the use of alternate metabolites (like glutamine). TME hypoxia is facilitated by this mechanism, which raises lactic acid, NO, reactive oxygen species, and other metabolic byproducts (57).

### 5.2 Continuous angiogenesis

Continuous angiogenesis in the TME is a multifaceted biological process involving various cell types and molecular pathways. This mechanism is critical for the growth and spread of tumors, as it provides them with the necessary supply of oxygen and nutrients (58). Prior research has established a substantial correlation between TAM infiltration levels and intratumor microvessel counts in NSCLC (59). Recent investigations have demonstrated that TAM can facilitate persistent angiogenesis in NSCLC through various pathways.

Xu et al. indicated that TAM can enhance the proliferation and migration of endothelial cells through the secretion of various angiogenic factors, including VEGF, FGF, and TGF-*β*, thereby facilitating the formation of new blood vessels (60). Chen et al. (61) observed that the interaction between TAM and tumor cells may up-regulate the expression of IL-8, which increases tumor angiogenesis in NSCLC patients to a great extent. Furthermore, TAM can facilitate matrix remodeling and augment angiogenesis by upregulating matrix metalloproteinases (62). In a hypoxic environment, TAM can activate hypoxia-inducible factor, which subsequently increases the production and secretion of angiogenic factors, thereby sustaining the tumor’s blood supply (63, 64).

### 5.3 The acquirement of the ability to infiltrate and transfer

The activation of epithelial-mesenchymal transition (EMT) is a critical mechanism in tumor cell metastasis, wherein epithelial cells adopt mesenchymal traits, resulting in increased motility and migration (65). EMT is characterized by the lack of epithelial cell markers and the increased expression of mesenchymal cell markers (66). Numerous studies have indicated that various cytokines and chemokines released by TAM might induce EMT.

A prior study indicated that TGF-*β* released by TAM can facilitate EMT and increase the expression of SOX9, hence augmenting the proliferation, migration, and invasion of NSCLC cells (67). Moreover, the suppression of TGF-β expression may impede EMT in NSCLC cells (68). Chen et al. (69) discovered that TAM generated from THP-1 exhibited elevated IL-6 expression when co-cultured with NSCLC cells, hence augmenting the invasive capacity of NSCLC cells through the modulation of EMT. Hu et al. (70) did a comparable investigation and discovered that IL-6 released by TAM can activate the JAK2/STAT3 pathway via autocrine signaling, with STAT3 functioning as a transcription factor to enhance the expression of C/EBP-*β*, so further increasing the transcription and production of IL-6. The establishment of a positive feedback loop involving IL6-STAT3-C/EBP-β-IL6 in TAM facilitates EMT and metastasis in LUAD (70). Suppression of EMT in NSCLC by the inhibition of M2-TAM mediated STAT3 signaling pathway (71). CXCL8 is a chemokine released by M2-TAM. Prior research indicated that CXCL8 may promote EMT and enhance the invasion and migration of NSCLC via the MAPK/NF-κB and JAK2/STAT3 signaling pathways (72, 73).

### 5.4 Evasion of immune surveillance

Immunosuppressive TME is the decisive element for cancer spread, immunological escape, and development of suppressed immune microenvironment. Immune cells are a crucial element of the immune system, regulating the equilibrium between inhibitory and cytotoxic responses in NSCLC (74). Research indicated that TAM can modulate immune surveillance by inhibiting the activity of other immune cells and attracting negative regulatory immune cells.

In the TME of NSCLC, TAM has been shown to enhance the expression of PD-L1, hence suppressing T cell cytotoxicity and phagocytosis, and facilitating T cell exhaustion by increasing the expression of IRF8. Furthermore, Young et al. observed that TAM also impedes the cytotoxicity of NK cells (75). Allavena et al. discovered that M2-TAM secreted immunosuppressive cytokines, such as IL-10 and TGF-*β*, within the TME, thereby diminishing the population of tumor-infiltrating lung dendritic cells and inhibiting their maturation (76). Regulatory T cells are a subset of T cells that inhibit the immunological response. CCL22 released by TAM promotes immunosuppressive TME by recruitment of Treg and suppresses the immunological function of CD8^+^ T cells, NK cells, B cells, and antigen-presenting cells (77).

### 5.5 Drug resistance treatment

One of the most popular and significant treatments for malignant tumors is chemotherapy. Tumor cells frequently become resistant to chemotherapy medications, much like bacteria are readily resistant to antibiotics. Numerous investigations have demonstrated the complex and significant role TAM plays in the development of treatment resistance in NSCLC. In the Lewis lung cancer (LLC) animal model, Hughes et al. discovered that chemotherapeutic drug treatment caused tumor cells to release CXCL12, which improved CD206^+^ TAM invasion, prevented tumor cell death, and aided tumor recurrence (78). By suppressing NEDD4L expression, the exosome miR-3679-5p released by TAM can indirectly stabilize the c-Myc protein, increasing aerobic glycolysis and ultimately fostering cisplatin resistance in NSCLC, according to Wang et al. (79). Furthermore, IL-6 or prostaglandin E2, which induces M2-TAM polarization by activating STAT3, STAT1, and STAT6 signaling pathways, can be secreted by tumor cells in response to cisplatin or carboplatin therapy. And cytotoxic chemotherapy resistance (80, 81).

As translational medicine has advanced, it has become clear that tumor-driven gene mutations use several signaling channel transduction processes to encourage the emergence and growth of malignancies. Although this finding makes tumor-focused therapy possible, likely, drug resistance issues will likely eventually arise with targeted therapy, and we are still working to find a solution (82). According to earlier research, NSCLC cells enhance their resistance to epidermal growth factor receptor-tyrosine kinase inhibitors (EGFR-TKIs) by promoting M2-TAM polarization and inhibiting M1-TAM polarization through the transfer of exosomes to TAM by targeting the miR-627-3p/Smads signaling pathway (83). A related work by Wang et al. (84) demonstrated that M2-TAM-generated exosomes regulated the MSTRG.292666.16/miR-6836-5p/MAPK8IP3 axis, hence promoting ocitinib resistance in NSCLC. Targeting TAM has also been demonstrated in studies to lessen acquired resistance to targeted therapy. By blocking the CD47-SIRRPα signal axis and M2 polarization in the co-culture system, Lu et al. discovered that STAT3 inhibitors can increase the phagocytic activity of TAM and decrease the acquired resistance to EGFR-TKIs. Furthermore, gefitinib reduced acquired resistance to gefitinib both *in vitro* and *in vivo* when combined with STAT3 inhibitors and anti-CD47 monoclonal antibodies (85).

Therefore, the anticancer activity of chemotherapeutic drugs and targeted agents may be enhanced when treatment is combined with intervention measures that decrease TAM infiltration or inhibit M2-TAM polarization.

## 6 The clinical importance of TAM

### 6.1 TAM and prognosis in NSCLC

Multiple studies have demonstrated that the level of TAM infiltration is directly associated with the prognosis of NSCLC patients (Table 2).

**Table 2:** The relationship between the infiltration, distribution, and classification of TAM and prognosis of NSCLC.

Tumor type	Markers	Prognosis	Article
NSCLC	CD68	Desirable prognosis: low TAM infiltration Undesirable prognosis: high TAM infiltration	Feng et al. (86)
NSCLC	CD68, TREM2	Desirable prognosis: low TREM2^+^ TAM infiltration Undesirable prognosis: high TREM2^+^ TAM infiltration	Zhang et al. (96)
NSCLC	CD68	Desirable prognosis: high TAM infiltration in the nest low TAM infiltration in the stromal Undesirable prognosis: low TAM infiltration in the nest high TAM infiltration in the stromal	Welsh et al. (97)
NSCLC	CD68	Desirable prognosis: high TAM infiltration in the nest low TAM infiltration in the stromal Undesirable prognosis: low TAM infiltration in the nest high TAM infiltration in the stromal	Kawai et al. (98)
NSCLC	CD68	Desirable prognosis: high TAM infiltration in the nest low TAM infiltration in the stromal Undesirable prognosis: low TAM infiltration in the nest high TAM infiltration in the stromal	Kim et al. (99)
NSCLC	CD68	Desirable prognosis: high TAM infiltration in the nest low TAM infiltration in the stromal Undesirable prognosis: low TAM infiltration in the nest high TAM infiltration in the stromal	Dai et al. (100)
NSCLC	M1: iNOS, HLA-DR, MRP 8/14, TNF-a M2: CD163, VEGF	Desirable prognosis: high M1 infiltration in the nest Undesirable prognosis: low M1 infiltration in the nest	Ohri et al. (101)
NSCLC	M1:CD68/HLA-DR M2:CD68/CD163	Desirable prognosis: high M1 infiltration in the nest Undesirable prognosis: low M1 infiltration in the nest	Ma et al. (87)
NSCLC	M1: CD68, iNOS M2: CD68, CD163	Desirable prognosis: high M1 infiltration in the nest low M2 infiltration in the nest and stromal Undesirable prognosis: low M1 infiltration in the nest high M2 infiltration in the nest and stromal	Jackute et al. (103)
NSCLC	M2: CD68, CD163	Desirable prognosis: low M2 infiltration in the nest Undesirable prognosis: high M2 infiltration in the nest	Cao et al. (104)
LUAD	M2: CD68, CD204	Desirable prognosis: low M2 infiltration Undesirable prognosis: high M2 infiltration	Ohtaki et al. (88)
LUAD	M1: CD68, iNOS M2: CD68, CD206	Desirable prognosis: low M2 infiltration Undesirable prognosis: high M2 infiltration	Zhang et al. (89)
LUAD	M2: CD204	Desirable prognosis: low M2 infiltration Undesirable prognosis: high M2 infiltration	Kaseda et al. (90)
LUSC	M2: CD204	Desirable prognosis: low M2 infiltration Undesirable prognosis: high M2 infiltration	Maeda et al. (91)
LUSC	M2: CD204	Desirable prognosis: low M2 infiltration Undesirable prognosis: high M2 infiltration	Hirayama et al. (92)
LUAD	M2: CD204	Desirable prognosis: low M2 infiltration Undesirable prognosis: high M2 infiltration	Sun and Xu (168)
NSCLC	M2: CD68, CD204	Desirable prognosis: low M2 infiltration Undesirable prognosis: high M2 infiltration	Li et al. (93)
NSCLC	M2: CD68, CD163	Desirable prognosis: low M2 infiltration Undesirable prognosis: high M2 infiltration	La Fleur et al. (94)
NSCLC	M2: CD68, CD163, VEGF-A, VEGF-C	Desirable prognosis: low M2 infiltration Undesirable prognosis: high M2 infiltration	Hwang et al. (95)
NSCLC	M1: CD68, HLA-DR; M2: CD68, CD204; M2: CD68, CD163	Desirable prognosis: high M1 infiltration in metastatic lymph node Undesirable prognosis: low M1 infiltration in metastatic lymph node	Rakaee et al. (102)
NSCLC	M2:CD163	Desirable prognosis: low infiltration of M2 in stromal and alveolar Undesirable prognosis: high infiltration of M2 in stromal and alveolar	Sumitomo et al. (105)

Research indicates that low TAM infiltration indicates a desirable prognosis, whereas high TAM infiltration is associated with an undesirable prognosis (86). For various TAM classifications, high M1-TAM infiltration typically indicates a desirable prognosis, whereas low M1-TAM infiltration is associated with an undesirable prognosis (87). Furthermore, low M2-TAM infiltration (88–95), and low TREM2^+^ TAM infiltration (96) are associated with a desirable prognosis.

Concerning the distribution of TAM in the TME, research indicates that high TAM infiltration in the nest and low TAM infiltration in the stromal typically indicate a desirable prognosis (97–100). Furthermore, high M1-TAM infiltration in the nest is typically correlated with a desirable prognosis (101). In addition, high M1-TAM infiltration in metastatic lymph nodes is regularly associated with a favorable prognosis (102). There is disagreement on the impact of M2-TAM distribution on prognosis. Jackute et al. indicated that low M2-TAM infiltration in both the nest and stroma is typically correlated with a desirable prognosis (103). Whereas Cao et al. (104) indicated that low M2-TAM infiltration in the nest is associated with a desirable prognosis, M2-TAM infiltration in the stroma is not correlated with the prognosis of NSCLC patients. In addition, low infiltration of M2-TAM in the stroma and alveoli correlated with a desirable prognosis (105).

These studies indicate that the infiltration, distribution, and classification of TAM considerably impact the prognosis of NSCLC patients and may serve as potential prognostic biomarkers.

### 6.2 TAM and responses to immunotherapy in NSCLC

Furthermore, research indicates that TAM is significantly associated with the response to immunotherapy in NSCLC. TREM2^+^ TAM is abundant in several anti-inflammatory cytokines and has an M2-type immunosuppressive phenotype, hence enhancing the inhibition of T-cell activity. TREM2^+^ TAM diminishes the anti-tumor efficacy of CD8^+^ T cells by exacerbating their malfunction and facilitating the development of FOXP3^+^ regulatory T cells. These alterations intensify the mechanism of immune evasion, enabling NSCLC cells to avoid elimination by the host immune system (96). The infiltration of TREM2^+^ TAM was significantly correlated with response rates to immunotherapy. Previous research revealed that patients undergoing PD-1-based immunotherapy with a low percentage of TREM2^+^ TAM in the TME typically exhibited a desirable treatment response rate, which indicates that TREM2^+^ TAM may adversely influence immunotherapy due to its immunosuppressive properties, thereby decreasing the effectiveness of PD-1 inhibitors (96). Decreasing the proportion of TREM2^+^ TAM in the TME may enhance the effectiveness of immunotherapeutic agents like PD-1 inhibitors.

### 6.3 Cytokines related to TAM and prognosis in NSCLC

The cytokines and chemokines secreted by TAM substantially influence the prognosis of NSCLC patients by regulating the tumor’s biological features (Table 3).

**Table 3:** The relationship between cytokines related to TAM and prognosis in NSCLC.

Tumor type	Prognosis	Marker	Article
NSCLC	Desirable prognosis: high IL-10 expression of TAM Undesirable prognosis: low IL-10 expression of TAM	IL-10	Wang et al. (106)
NSCLC	Desirable prognosis: low YKL-40 expression of TAM Undesirable prognosis: high YKL-40 expression of TAM	YKL-40	Thöm et al. (108)
NSCLC	Desirable prognosis: low IL-34 and M-CSF expression of TAM Undesirable prognosis: high IL-34 and M-CSF expression of TAM	IL-34 and M-CSF	Baghdadi et al. (107)

IL-10, as an immunosuppressive cytokine, can impede the anti-tumor immune response, facilitate tumor immune evasion, and hence expedite tumor development. The research has indicated that upregulated expression of IL-10 in TAM is significantly associated with poorly differentiated NSCLC (106). IL-34 serves as a ligand for the CSF-1R, facilitating the recruitment of TAM and triggering M2-TAM polarization. Baghdadi et al. discovered that in patients with advanced NSCLC, IL-34, by its interaction with CSF-1R, facilitates the M2-TAM polarization, which is significantly associated with tumor immunosuppression and tumor growth (107). Moreover, serum YKL-40 levels have been significantly correlated with the prognosis of NSCLC. Elevated YKL-40 levels are regarded as an independent predictive indicator of worse survival in individuals with metastatic NSCLC. YKL-40 is a glycoprotein that influences the immunological microenvironment of tumors and facilitates their aggressive growth through interactions with immune cells (108). Consequently, the identification of YKL-40 serves as an indicator for assessing the course of NSCLC and may potentially offer significant guidance for personalized treatment (108).

These molecular markers offer novel potential targets for the early diagnosis and prognostic evaluation of NSCLC, along with prospective avenues for future immunotherapy therapies.

## 7 TAM as the potentially therapeutic target for NSCLC

In recent years, the rapid progress in molecular biology and tumor immunology has established drug research targeting TAM as a novel emphasis in cancer treatment. Researchers have discovered that various Chinese herbal formulas and natural compounds could target TAM as a potential treatment for NSCLC. The advancement of novel nanomaterials can enhance medication accumulation at tumor sites by targeting TAM, while simultaneously minimizing systemic toxicity (109). Additionally, TAM plays a critical role in influencing the effectiveness of radiotherapy, chemotherapy, immunotherapy, and targeted therapy in NSCLC.

### 7.1 Natural compounds

Natural compounds exhibit promise in the management of NSCLC by targeting TAM. Numerous natural substances possess anti-tumor activities and can enhance therapeutic outcomes by regulating the function of TAM. Presented are many promising natural compounds together with their modes of action (Table 4).

**Table 4:** Natural compounds formulas as the potential treatment for NSCLC by targeting TAM.

Classification	Name	Function	Mechanism
Natural compound	Dihydroartemisinin	Induce M2 macrophages to polarize into M1 type	Initiate the Akt/mTOR signaling pathway (133)
Natural compound	Dioscin	Induce M2 macrophages to polarize into M1 type	Downregulating IL-10 expression, activating the JNK signaling pathway, and blocking the STAT3 signaling pathway (114)
Natural compound	Ginsenoside Rh2	Induce M2 macrophages to polarize into M1 type	The expression of the M2 macrophage marker CD206 was diminished, the expression of the M1 macrophage markers CD16/32 was elevated, and the expression levels of VEGF, MMP2, and MMP9 were reduced (115).
Natural compound	Curcumin	Augment the polarization of M1 macrophages	Increased expression of IL-6 and TNF-α (143)
Natural compound	Puerarin	Induce M2 macrophages to polarize into M1 type	The expressions of IL-10, IL-4, and TGF-β were decreased, whereas the expressions of IFN-γ, TNF-α, and IL-12 were elevated (130).
Natural compound	Astragalus polysaccharides	Induce M2 macrophages to polarize into M1 type	Decreased the expression of IL-4 and IL-13, increased the expression of LPS and IFN-γ (132).
Natural compound	Astragaloside IV	Suppression of M2 macrophage polarization	Decreased expression of IL-13 and IL-4 (113)
Natural compound	Paeoniflorin	Suppression of M2 macrophage polarization	Decreased expression of IL-4 (116)
Natural compound	Matrine	Suppression of M2 macrophage polarization	Suppression of the PI3K/Akt/mTOR signaling pathway (122)
Natural compound	Resveratrol	Suppression of macrophage recruitment and suppression of M2 macrophage polarization	F4/80^+^ cells diminished and suppressed STAT3 phosphorylation (128)
Natural compound	Sanguinarine	Suppression of M2 macrophage polarization	Suppression of the WNT/β-Catenin signaling pathway (120)
Natural compound	Dihydroisotanshinone I	Suppression of macrophage recruitment	Suppression of the CCL2/STAT3 signaling pathway and reduction of CCL2 release (136)
Natural compound	Polyphyllin VII	Augment the polarization of M1 macrophages	Initiate the STING/TBK1/IRF3 signaling pathway and suppress STAT3 phosphorylation (119)
Natural compound	Sophoridine	Augment the polarization of M1 macrophages	Initiate the MARK signaling pathway, enhance the expression of IFN-g, TNF-a, IL-6, iNOS, and IL-1b, and elevate the expression of CD86 on the surface of the M1 macrophage marker (121).
Natural compound	Ginseng Berry Polysaccharides Portion	Augment the polarization of M1 macrophages	Increased expression of IL-6, IL-12, and TNF-α (134)

#### 7.1.1 Steroidal saponins

Steroid saponins are saponins that exist in an unbound form. *In vitro* and *in vivo* investigations have demonstrated that steroid saponins exhibit extensive anti-tumor properties, including the inhibition of tumor cell proliferation, induction of tumor cell apoptosis and autophagy, as well as the suppression of tumor invasion and metastasis (110–112), characterized by low toxicity and high anti-tumor efficacy. The study elucidates the mechanism via which steroid saponins modulate NSCLC by targeting TAM. Xu et al. discovered that the infiltration of M2-TAM in neoplastic tissues diminished following Astragalus IV administration, significantly suppressing tumor proliferation (113). Cui et al. (114) demonstrated that diosgenin can stimulate anti-tumor immunity in NSCLC by decreasing IL-10 secretion from TAM in the TME, modulating STAT3 and JNK signaling pathways, and facilitating the polarization of M2-TAM to M1-TAM. Li et al. (115) discovered that ginsenoside Rh2 diminishes the expression of the M2-TAM marker CD206, enhances the expression of the M1 marker CD16/32, decreases the levels of VEGF, MMP2, and MMP9, facilitates the reprogramming of TAM, and polarises them from M2-TAM to M1-TAM. Inhibiting the migration of NSCLC cells. Paeoniflorin has been demonstrated to impede NSCLC metastasis by down-regulating IL-4 production and blocking the polarization of M2-TAM (116). Zonoside VII is an active monomer of the genus Zonoside, demonstrating significant anti-tumor efficacy across multiple tumor types (117, 118). Yu et al. discovered that in the TME of NSCLC, regonoside VII can block STAT3 phosphorylation by activating the STING/TBK1/IRF3 pathway, promote the polarization of M1-TAM, and potentially up-regulate PD-L1 expression. Thus improving the effectiveness of immune checkpoint inhibitors (119).

#### 7.1.2 Alkaloids

Alkaloids impede the proliferation and metastasis of NSCLC cells through the modulation of TAM polarization. Matrine, sophoridine, and sanguinarine are the most prominent among them. Cui et al. (120) discovered that sanguinarine can target the WNT/*β*-Catenin pathway, suppress polarization of M2-TAM, and have anti-angiogenic actions on NSCLC, along with the modulation of immunological factors. Zhao et al. (121) demonstrated that sophoidine enhances the secretion of pro-inflammatory cytokines IFN-*γ*, TNF-*α*, IL-6, iNOS, and IL-1β by activating the MARKs signaling pathway, and upregulates the expression of the M1-TAM surface marker CD86. It can facilitate the polarization of M1-TAM and impede the proliferation of NSCLC. Matrine has been shown to down-regulate the expression levels of IL-4, Arg-1, and IL-10 by blocking the PI3K/Akt/mTOR signaling pathway, consequently suppressing the polarization of M2-TAM and the metastasis of NSCLC (122).

#### 7.1.3 Polyphenols

Polyphenols are compounds characterized by benzene rings and multiple hydroxyl groups in their chemical structure, capable of modulating various signal transduction pathways, including PI3K/Akt, MAPK, and NF-κB, which are crucial in tumor growth, proliferation, and metastasis (123, 124). Curcumin and resveratrol have garnered significant interest. Curcumin exhibits several biological effects, including anti-inflammatory, anti-tumor, and antioxidant properties. Due to its reduced adverse effects, curcumin has been utilized by several researchers as an anti-tumor agent (125, 126). Wang et al. (127) discovered that curcumin enhances the secretion of IL-6 and TNF-*α* in the TME of NSCLC, reprograms M2-TAM to tumoricidal M1-TAM, and creates an innovative nanomedical approach for combination therapy of NSCLC. Sun et al. discovered that the F4/80 positive cells in the TME of LCC mice treated with resveratrol diminished, suggesting that resveratrol may impede the recruitment of TAM, thereby decreasing their infiltration. Moreover, resveratrol has demonstrated the capacity to impede the activation and differentiation of M2-TAM by blocking STAT3 phosphorylation (128).

#### 7.1.4 Additional categories

Puerarin is an isoflavone molecule derived from Pueraria, exhibiting numerous pharmacological activities including vasodilation, heart protection, neuroprotection, anticancer effects, antioxidant properties, and anti-inflammatory actions (129). Puerarin greatly enhances the expression of anti-tumor cytokines IFN-*γ*, TNF-*α*, and IL-12, while diminishing the levels of anti-inflammatory cytokines IL-10, IL-4, and TGF-*β*, thereby inhibiting the polarization of M2-TAM and promoting the polarization of M1-TAM (130). Astragalus polysaccharide is a polysaccharide derived from Astragalus, which has garnered significant attention in cancer treatment research in recent years (131). Bamodu et al. established that Astragalus polysaccharide facilitates the polarization of M2-TAM to M1-TAM by down-regulating IL-10, IL-4, and TGF-β while up-regulating IFN-*γ*, TNF-*α*, and IL-12, so augmenting the anti-cancer immune response (132). The artemisinin derivative dihydroartemisinin has demonstrated the capacity to diminish TAM infiltration in the TME of LLC mice via the Akt/mTOR signaling pathway, drastically elevate the M1/M2 ratio of TAM, and augment the phagocytic capability of M1-TAM (133). Furthermore, in murine studies, Lee et al. established that ginseng berry polysaccharide partially elicited a pro-immune response by augmenting the expression of IL-6, IL-12, and TNF-α in mouse peritoneal TAM, and prompted the polarization of TAM towards the M1 phenotype, thereby emerging as a potential therapeutic agent for immunotherapy in NSCLC (134). Dihydrotanshinone I is a lipophilic terpenoid molecule, which is considered an anticancer agent. It has been documented to impede the proliferation of certain cancer types (135). The fundamental mechanism of NSCLC remains ambiguous. Wu et al. established that dihydrotanshinone I can obstruct the CCL2/STAT3 signaling pathway, diminish CCL2 release from TAM and NSCLC cells, and impede the TAM recruitment capacity of NSCLC cells (136).

### 7.2 Chinese herbal formula

Several clinical studies have commenced investigating the efficacy of traditional Chinese medicine formulations in patients with NSCLC (Table 5), and initial findings indicate that traditional Chinese medicine may enhance patients’ quality of life and maybe improve outcomes. Wang et al. (137) discovered that Yupingfeng Powder facilitated M1-TAM polarization by enhancing STAT1 phosphorylation, activating CD4^+^ T cells, augmenting their cytotoxicity, and suppressing the *in situ* development of LLC. Moreover, Bu-Fei decoction, a traditional Chinese medicine formulation, can impede the proliferation, migration, invasion, and immunosuppression of M2-TAM-generated NSCLC by suppressing the production of IL-10 and PD-L1 (138). Jinfu’an decoction facilitates the conversion of M2-TAM to M1-TAM, amplifies the anticancer efficacy of cisplatin, and diminishes the expression of markers associated with M2-TAM. This regulatory action may be associated with the downregulation of *β*-catenin expression (139). Research on Traditional Chinese Medicine (TCM) aimed at targeting TAM is still developing, necessitating additional clinical trials in the future to validate the effectiveness of TCM formulations and their underlying processes. Simultaneously, in conjunction with contemporary biotechnology, novel active compounds and targets may be identified to facilitate a more efficacious treatment for NSCLC.

**Table 5:** Chinese herbal formulas as the potential treatment for NSCLC by targeting TAM.

Classification	Name	Function	Mechanism
Chinese herbal formula	Bu-Fei decoction	Suppression of M2 macrophage polarization	Decreased the expression of PD-L1 and IL-10 (138)
Chinese herbal formula	Yu-Ping-Feng decoction	Augment the polarization of M1 macrophages	Increased phosphorylation of STAT1 (137)
Chinese herbal formula	Jinfu’an decoction	Induce M2 macrophages to polarize into M1 type	Decreased expression of β-catenin (139)

### 7.3 Nanomedications

Nanomedicine is an innovative product resulting from the integration of nanotechnology and medicine. Increasing emphasis is being directed toward novel pharmaceuticals for the diagnosis, treatment, and prevention of numerous ailments, including tumors and immunological disorders.

Liposomes represent a principal category of anti-tumor nanomedicine and are among the most successful in clinical application (140). Li et al. (141) engineered a folate-modified liposome nanoparticle for the targeted delivery of PFH, STAT3 siRNA, and Fe3O4 to the TME. The dual effect prevented the polarization of M2-TAM in the TME and facilitated their transition into M1-TAM, hence increasing T cell activation and proliferation, which enhanced the immunological response to NSCLC. Tie et al. (142) developed folate-modified liposomes as BIM-S plasmid vectors, which markedly enhanced apoptosis in tumor cells and M2-TAM by specifically targeting NSCLC cells, and substantially suppressed tumor growth *in vivo*. Zhu et al. (143) created an innovative carboxymethyl chitosan (CMCS)-based nanoparticle (CBT-DC) that targets transferrin receptors on NSCLC cells and accurately modulates the release of docetaxel and curcumin in response to pH and reactive oxygen species (ROS) levels. The findings indicated that CBT-DC had superior anti-tumor efficacy both *in vitro* and in vivo compared to docetaxel monotherapy and other nanocarriers containing just docetaxel and curcumin. Su et al. (144) developed nanovesicles utilizing microfluidic technology for the delivery of CD47/PD-L1 antibodies. It can only be liberated upon dissociation in the acidic tumor milieu, thereby ameliorating immune-related adverse events, such as anemia, pneumonia, hepatitis, and small intestinal inflammation caused by off-target effects, and facilitating NSCLC-specific activation immunotherapy. Furthermore, Wang et al. (127) developed nanovesicles utilizing oligomeric hyaluronic acid polymers to encapsulate curcumin and baicalin. *In vitro* cellular assays and *in vivo* anti-tumor studies on A549 tumor-bearing mice showed that the carrier material targeting nano micelles exhibits significant cytotoxicity and cellular penetration, effective anti-tumor efficacy, and minimal adverse effects. Nanomedicine aimed at TAM offers an innovative approach to the management of NSCLC. By precisely targeting and regulating TAM, nano-agents are anticipated to enhance anti-tumor immune responses and overall treatment effectiveness. The subsequent study ought to concentrate on refining the design and clinical utilization of nanomaterials to enhance therapeutic results.

### 7.4 TAM and NSCLC treatment

Conventional therapies for NSCLC mostly encompass radiotherapy, chemotherapy, immunotherapy, and targeted therapy. The combination of targeted TAM therapy with conventional treatments can more effectively modulate the tumor microenvironment and enhance therapeutic outcomes, particularly in patients with advanced or metastatic NSCLC.

#### 7.4.1 TAM and radiotherapy

Radiotherapy employs high-energy radiation to obliterate the DNA of tumor cells, thereby impeding their proliferation and reproduction. It can directly eradicate tumor cells while also indirectly enhancing the anti-tumor immune response by stimulating the immune system (145). Nonetheless, radiotherapy is recognized for its restricted clinical effectiveness in lung cancer patients owing to tumor resistance to radiation and the necessity to escalate radiation doses. A significant component contributing to this limitation is the immunosuppressive microenvironment, where immunosuppressive cells assist tumor cells in evading radiation (146, 147).

A recent study revealed that RT upregulates STAT6 signaling pathways that facilitate the polarization and aggregation of M2-TAM in NSCLC. Inhibiting the STAT6 signaling pathway can diminish the population of M2-TAM and facilitate their reprogramming to the Mz1 phenotype, hence increasing the susceptibility of NSCLC to radiotherapy. Moreover, inhibiting STAT6 downregulates TGF-*β* levels and amplifies anti-tumor efficacy. The amalgamation of STAT6 inhibitors and radiotherapy can impede the proliferation of both primary and metastatic tumors in NSCLC (148).

Reprogramming from the M2-TAM to the M1-TAM, along with the upgradation of cytotoxic T cell activity through ICIs and other inhibitors of immunosuppressive factors, can augment the immunological response to radiotherapy and synergistically amplify its anticancer efficacy. This is anticipated to decrease the necessary dosage of radiotherapy to mitigate systemic damage (149, 150).

#### 7.4.2 TAM and chemotherapy

The resistance of tumor cells to chemotherapy presents a substantial obstacle in cancer treatment, particularly in NSCLC, where this resistance markedly influences therapeutic efficacy (151, 152). Research indicated that the accumulation and polarization of M2-TAM significantly contribute to chemotherapy resistance as a facilitator of tumor growth and immunosuppression via many pathways (153, 154).

IL-34, a ligand for CSF-1R, is recognized for its capacity to enhance the viability, growth, differentiation, and proliferation of monocytes and macrophages. Prior research has demonstrated that in NSCLC, IL-34 secreted by tumor cells can modulate the activity of TAM through AKT signaling activation, hence augmenting local immunosuppression and facilitating the survival of chemotherapy-resistant tumor cells. Further research shows that inhibiting IL-34 in chemotherapy-resistant tumors markedly suppresses tumor proliferation (155). P2X7, as an essential sensor of extracellular ATP, is extensively present in various immune cells and serves as a potent inflammatory and immunological activator (156). The expression of P2X7 was upregulated in TAM (157). Prior research indicated that P2X7 deficiency can inhibit M2-TAM polarization by down-regulating STAT6 and IRF4 phosphorylation. Furthermore, P2X7 deficiency enhances T-cell mobilization, reverses M2-TAM polarization, and curtails the advancement of NSCLC by diminishing tumor cell proliferation and angiogenesis. Consequently, the inhibition or obstruction of P2X7 yields a therapeutic benefit in NSCLC. Subsequent research indicated that the coadministration of the P2X7 inhibitors O-ATP, A-438079 hydrochloride, and A-740003 mitigated resistance to cisplatin (158).

The aforementioned results demonstrate that by targeting TAM and its regulated immunosuppressive mechanisms, immune evasion, and chemotherapy resistance in tumors can be effectively overcome, hence enhancing the overall therapeutic efficacy. This discovery offers a novel approach to the treatment of NSCLC.

#### 7.4.3 TAM and immunotherapy

Immunotherapy has emerged as a crucial treatment for NSCLC by stimulating the patient’s immune system to identify and eliminate tumor cells, with ICIs such as PD-1/PD-L1 and CTLA-4 inhibitors being integral to this mechanism (159). The significance of TAM in NSCLC immunotherapy has garnered considerable attention in recent years. Leucine-rich repeat-containing G-protein-coupled receptor 4 (Lgr4) is identified as a crucial regulator of TAM polarization. The polarization of M2-TAM can be enhanced via the Rspo/Lgr4/Erk/Stat3 signaling pathway, and inhibiting this route can mitigate lung cancer’s resistance to PD-1 therapy and enhance therapeutic efficacy (160). The transcription factor c-Maf serves as a metabolic checkpoint that regulates the TCA cycle and UDP-GlcNAc production, hence facilitating the polarization and activation of M2-TAM. Liu et al. (161) established the LLC mice model and discovered that c-Maf inhibition partially mitigates resistance to PD-1 treatment. In conclusion, targeting TAM and controlling their polarization can markedly enhance the effectiveness of lung cancer immunotherapy. Strategies involving the inhibition of particular receptors and the regulation of TAMs are anticipated to address drug resistance in immunotherapy.

#### 7.4.4 TAM and targeted therapy

For NSCLC patients possessing identifiable driver-sensitive mutations, targeted therapy has emerged as the standard of care. The mutation of the EGFR is a significant catalyst for NSCLC. EGFR-TKIs represent a significant advancement in lung cancer treatment, demonstrating improved efficacy compared to standard chemotherapy in patients with advanced EGFR mutation-positive NSCLC. A recent study has demonstrated that EGFR-TKIs can target TAM for NSCLC patients, hence augmenting anti-tumor efficacy. Tariq et al. (162) discovered that gefitinib can inhibit IL-13-induced phosphorylation of STAT6, which was a crucial signaling pathway in M2-TAM polarization. Imatinib (163) and Lapatinib (164) have demonstrated analogous modes of action in inhibiting M2-TAM polarization. These findings indicated that EGFR-TKIs may dynamically modify the cellular composition of TME in NSCLC patients. Aligning immune-stimulating conditions with targeted therapies can influence the enduring efficacy for patients because of the intricate interactions between macrophages in the TME and targeted medications (165–167).

In conclusion, TAM plays a critical role in influencing the effectiveness of radiotherapy, chemotherapy, immunotherapy, and targeted therapy in NSCLC, with strategies targeting TAM polarization and its immunosuppressive mechanisms enhancing treatment efficacy and overcoming resistance.

## 8 Perspective

In NSCLC research, TAM is increasingly acknowledged as a crucial immunomodulator. This study examines the significant significance of TAM in the progression of NSCLC, highlighting its intricate involvement in the TME, which influences tumor growth and metastasis, as well as profoundly affecting the immune response in NSCLC patients. The study indicates that TAM can profoundly influence the clinical manifestations and patient outcomes of NSCLC via metabolic reprogramming, persistent angiogenesis, enhanced infiltration, and metastatic potential, evasion of immune surveillance, and the facilitation of therapeutic resistance. Numerous clinical drugs, Chinese herbal formulas, and natural compounds have exhibited efficacy in targeting TAM for the treatment of NSCLC. Additionally, the advancement of novel nanomedicine offers a fresh perspective for the precise treatment of NSCLC.

Future TAM research is anticipated to achieve significant advancements in various areas. The focused therapy method for TAM will be a significant research focus. By identifying distinct surface markers and signaling pathways, researchers can create innovative tailored therapeutics to modify the activity of TAM, thereby augmenting the anti-tumor immune response. Secondly, the ongoing progress in scRNAseq technology enables researchers to investigate the heterogeneity of TAM and its function across various tumor types more comprehensively. This will establish a theoretical foundation for individualized treatment. Furthermore, the integration of nanomedicine technology with the creation of tumor-targeted nanomedicine based on TAM may emerge as a significant research avenue in the future, enhancing medication targeting while minimizing harm to normal tissues. Ultimately, interdisciplinary collaboration and research will advance the area of TAM, enhance the integration of fundamental research with clinical application, and offer more effective therapy alternatives for NSCLC patients.
